# Supplementary material for: Systemic Inflammation, Endothelial Function, and Risk of Periodontitis in Overweight/Obese Adults
Source: Biomedicines. 2023 May 23;11(6):1507. doi: 10.3390/biomedicines11061507 (PMC10294979; doi:10.3390/biomedicines11061507)
Supplement: Supplementary file 1 [file biomedicines-11-01507-s001.zip › biomedicines-2366359-supplementary.pdf]

Figure S1: Diagram of theoretical interrelationship between the biomarkers and their associations with periodontal disease (PD) according to literature.

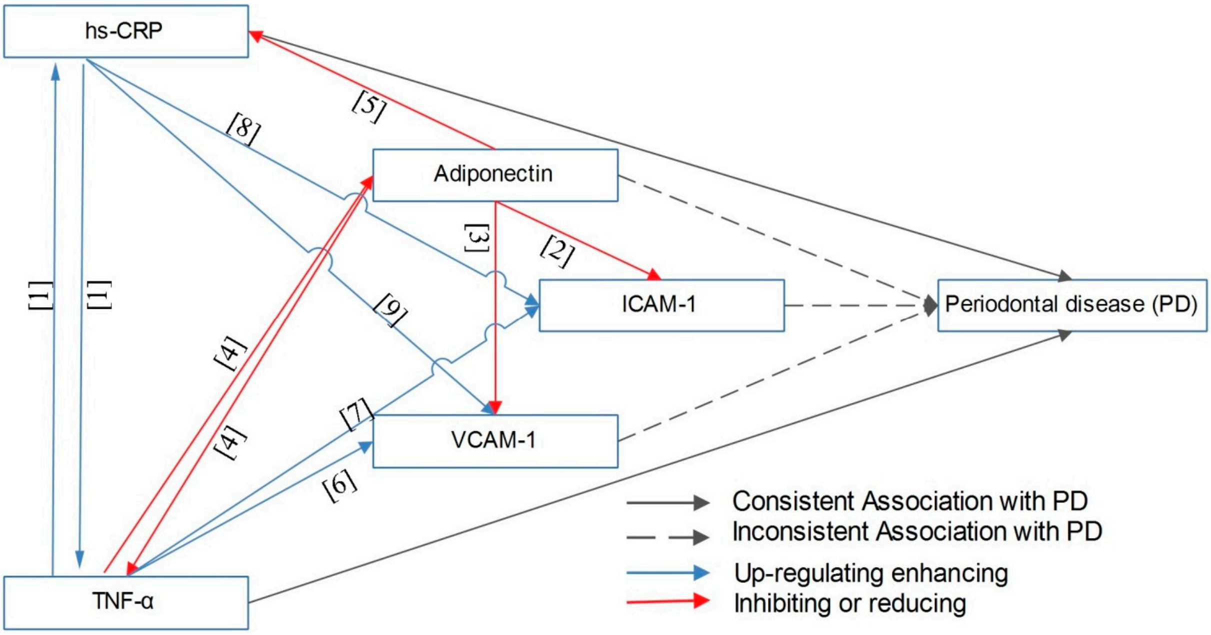

References for the network of inflammatory mediators:

Arrows: [1] TNF-α and hs-CRP [1]; [2] Adiponectin and ICAM-1[2]; [3] Adiponectin and VCAM-1[2]; [4] Adiponectin and TNF-α[2-3]; [5] Adiponectin and hs-CRP[3]; [6] TNF-α and VCAM-1[4]; [7] TNF-α and ICAM-1[4-5]; [8] hs-CRP and ICAM-1[6]; [9] hs-CRP and VCAM-1[6]

Figure S2. Generalized structural equation (GSEM) models of the interrelationship between the biomarkers (with 1 standard deviation increase: $\beta_{\Delta=+SD}$)†, including ICAM-1, and the outcome number of teeth with PPD ≥ 4mm and BOP (N=597).

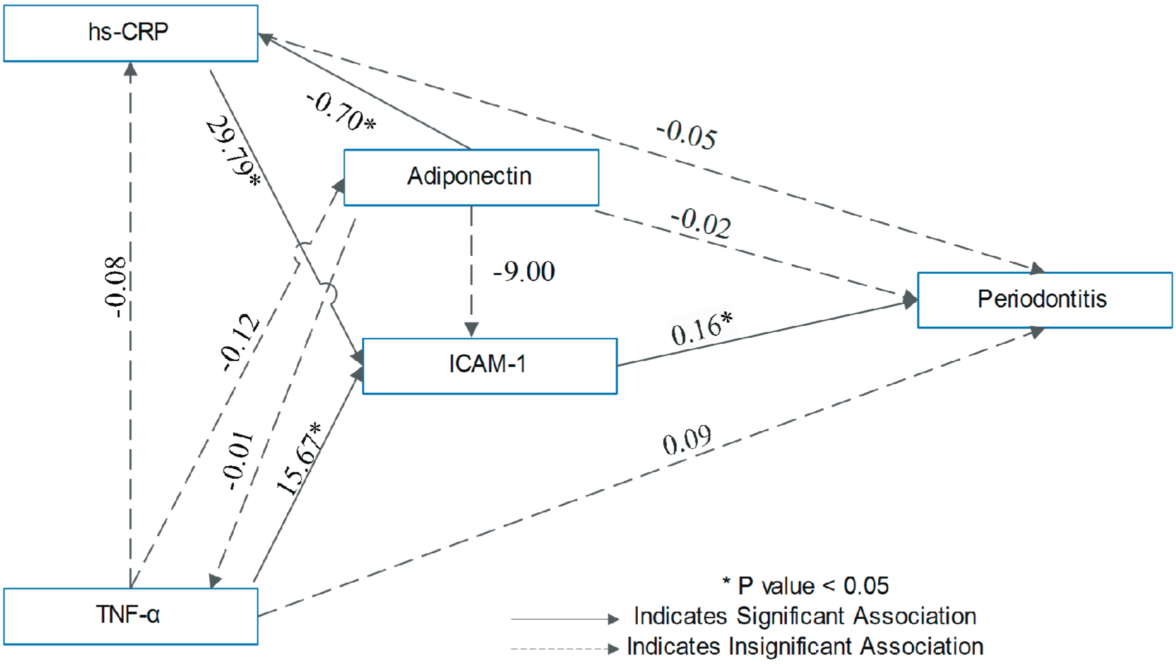

† Numbers in the figure indicates beta coefficient ($\beta_{\Delta=+SD}$) of direct association between two connected variables in reference to number of teeth with PPD ≥ 4mm and BOP, after adjusting covariates under GSEM-negative binominal regression model.

Figure S3. Generalized structural equation (GSEM) models of the interrelationship between the biomarkers (with 1 standard deviation increase: $\beta_{\Delta = +SD}$)†, including VCAM-1, and the outcome number of teeth with PPD ≥ 4mm and BOP (N=597).

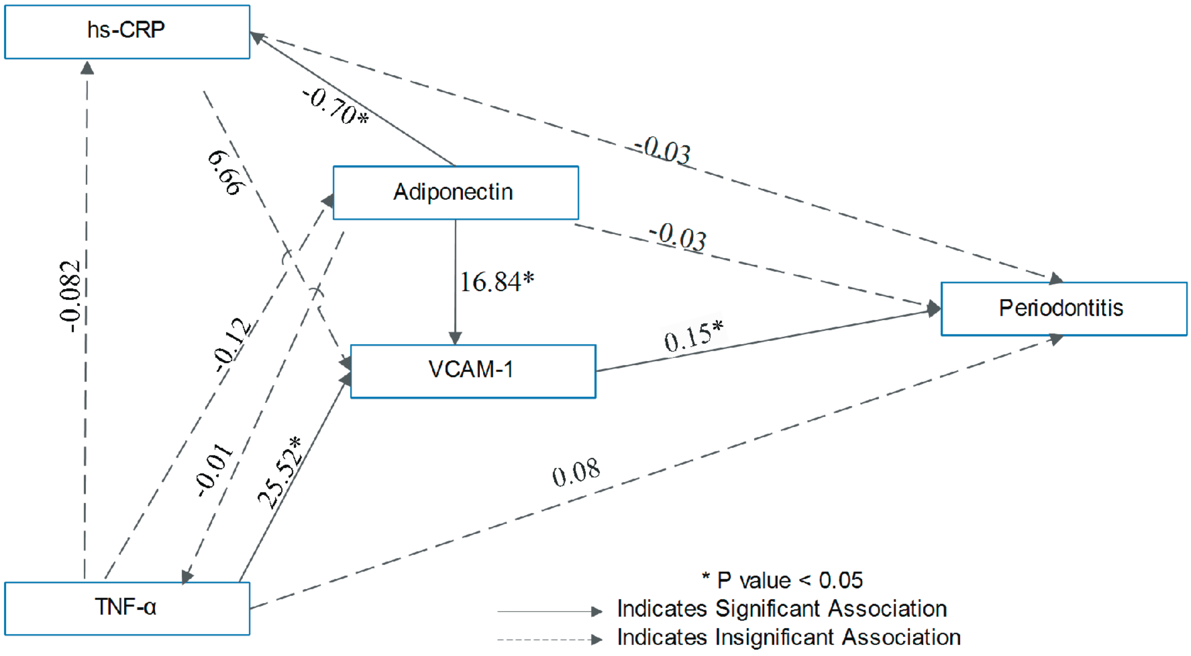

† Numbers in the figure indicates beta coefficient ($\beta_{\Delta = +SD}$) of direct association between two connected variables in reference to number of teeth with PPD ≥ 4mm and BOP, after adjusting covariates under GSEM-negative binominal regression model.

References:

1. (a) Ebersole, J. L.; Cappelli, D., Acute-phase reactants in infections and inflammatory diseases. *Periodontology 2000* **2000,** *23*, 19-49; (b) Ballou, S. P.; Lozanski, G., Induction of inflammatory cytokine release from cultured human monocytes by C-reactive protein. *Cytokine* **1992,** *4* (5), 361-8.

2. Ouchi, N.; Walsh, K., Adiponectin as an anti-inflammatory factor. *Clin Chim Acta* **2007,** *380* (1-2), 24-30.

3. Koleva, D. I.; Orbetzova, M. M.; Nikolova, J. G.; Deneva, T. I., Pathophysiological Role of Adiponectin, Leptin and Asymmetric Dimethylarginine in the Process of Atherosclerosis. *Folia Med (Plovdiv)* **2016,** *58* (4), 234-240.

4. Davies, M. J.; Gordon, J. L.; Gearing, A. J.; Pigott, R.; Woolf, N.; Katz, D.; Kyriakopoulos, A., The expression of the adhesion molecules ICAM-1, VCAM-1, PECAM, and E-selectin in human atherosclerosis. *J Pathol* 1993, 171 (3), 223-9.

5. Hosokawa, Y.; Hosokawa, I.; Ozaki, K.; Nakae, H.; Matsuo, T., Cytokines differentially regulate ICAM-1 and VCAM-1 expression on human gingival fibroblasts. Clin Exp Immunol 2006, 144 (3), 494-502.

6. Pasceri, V.; Willerson, J. T.; Yeh, E. T., Direct proinflammatory effect of C-reactive protein on human endothelial cells. Circulation 2000, 102 (18), 2165-8.
